# SIX2 promotes cell plasticity via Wnt/β-catenin signalling in androgen receptor independent prostate cancer

**DOI:** 10.1093/nar/gkae206

**Published:** 2024-03-30

**Authors:** Noora Leppänen, Heidi Kaljunen, Eerika Takala, Roosa Kaarijärvi, Petri I Mäkinen, Seppo Ylä-Herttuala, Ilkka Paatero, Ville Paakinaho, Kirsi Ketola

**Affiliations:** Institute of Biomedicine, University of Eastern Finland, Kuopio, Finland; Institute of Biomedicine, University of Eastern Finland, Kuopio, Finland; Institute of Biomedicine, University of Eastern Finland, Kuopio, Finland; Institute of Biomedicine, University of Eastern Finland, Kuopio, Finland; A.I. Virtanen Institute, University of Eastern Finland, Kuopio, Finland; A.I. Virtanen Institute, University of Eastern Finland, Kuopio, Finland; Heart Center and Gene Therapy Unit, Kuopio University Hospital, Kuopio, Finland; Turku Bioscience Centre, University of Turku and Åbo Akademi University, Turku, Finland; Institute of Biomedicine, University of Eastern Finland, Kuopio, Finland; Institute of Biomedicine, University of Eastern Finland, Kuopio, Finland

## Abstract

The use of androgen receptor (AR) inhibitors in prostate cancer gives rise to increased cellular lineage plasticity resulting in resistance to AR-targeted therapies. In this study, we examined the chromatin landscape of AR-positive prostate cancer cells post-exposure to the AR inhibitor enzalutamide. We identified a novel regulator of cell plasticity, the homeobox transcription factor SIX2, whose motif is enriched in accessible chromatin regions after treatment. Depletion of SIX2 in androgen-independent PC-3 prostate cancer cells induced a switch from a stem-like to an epithelial state, resulting in reduced cancer-related properties such as proliferation, colony formation, and metastasis both *in vitro* and *in vivo*. These effects were mediated through the downregulation of the Wnt/β-catenin signalling pathway and subsequent reduction of nuclear β-catenin. Collectively, our findings provide compelling evidence that the depletion of SIX2 may represent a promising strategy for overcoming the cell plasticity mechanisms driving antiandrogen resistance in prostate cancer.

## Graphical Abstract

**Graphical Abstract:**
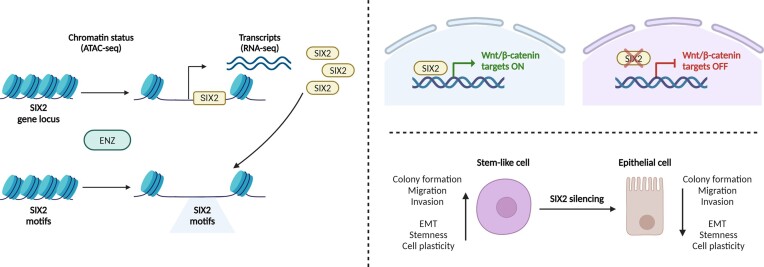

## Introduction

Resistance to potent inhibitors of the androgen receptor (AR) pathway, such as enzalutamide (ENZ) and abiraterone, poses a significant challenge in the treatment of advanced prostate cancer, ultimately resulting in therapy failure (1,2). Lineage plasticity including activation of stem cell transcriptional program is encountered in a subset of treatment resistant prostate tumors (3–8). Current understanding of prostate cancer treatment resistance highlights extensive transcriptional reprogramming during the evolution of castration-resistant prostate cancer (CRPC) to lineage plastic phenotypes (9–11). However, the identification of key phenotype-defining master transcription factors and early drivers of the lineage plasticity post AR inhibition therapies leading to resistant cell fates in prostate cancer remains unclear.

In this study, we investigated changes in chromatin accessibility and potential androgen receptor-suppressed genes under antiandrogen treatment to identify key factors driving resistant phenotypes in prostate cancer. Through the analysis of chromatin accessibility and open chromatin regions following antiandrogen ENZ treatment, we identify Sine oculis homeobox 2 (SIX2), a developmental transcription factor, as a key phenotype-defining transcriptional regulator of stem cell transcriptional program highly expressed under antiandrogen therapy. Our data reveals that chromatin sites containing the SIX2 binding motif become accessible following ENZ treatment. The loss of SIX2 alters the activity of Wnt/β-catenin signalling and reduces nuclear β-catenin, resulting in reduced pluripotency of PC-3 cells. In addition, SIX2 depletion induced mesenchymal to epithelial transition, and decreased metastatic properties such as migration, invasion, and colony formation both *in vitro* and *in vivo*.

SIX2 is an evolutionarily conserved transcription factor containing a DNA binding homeodomain. SIX2 plays a central role within the kidney mesenchyme by promoting cell proliferation and self-renewal, as well as maintaining multipotency of the nephron progenitor population during embryogenesis (12–14). While SIX2 has been shown to enhance the cell stemness in non-small cell lung cancer (15) and promote the expression of embryonic stem cell-associated programs, metastatic progression and the expression of key pluripotency factors *SOX2* and *NANOG* in triple-negative breast cancer (16,17), its role in prostate cancer remains unknown. Here, we demonstrate that SIX2 expression is significantly upregulated in antiandrogen-induced resistance. Moreover, we propose that inhibiting SIX2 could potentially prevent the development of treatment-induced resistant phenotypes and reduce the metastatic potential of prostate cancer cells, both *in vitro* and *in vivo*.

## Materials and methods

### Cell lines

AR-positive LNCaP and 22Rv1, and AR-negative PC-3, DU145 and NCI-H660 cells were utilized in this study. LNCaP, 22Rv1, PC-3 and DU145 cells were cultured in RPMI 1640 Dulbecco's medium, supplemented with 10% fetal bovine serum (FBS), a combination of 100 U/ml penicillin and 100 μl/ml streptomycin (Gibco Pen Strep, Thermo Fisher Scientific) and 2 mM l-glutamine. NCI-H660 cells were maintained in RPMI 1640 Dulbecco's medium containing 5% FBS, 10 μg/ml transferrin, 30 nM sodium selenite, 10 nM β-estradiol, 2 mM l-glutamine, 5 μg/ml insulin, and 10 nM hydrocortisone. For cells exposed to enzalutamide (ENZ), the culture medium was supplemented with 10 μM ENZ (Orion, ORM-0016678). DMSO (D8418-250ML, Merck) was used as control for ENZ. Mycoplasma assessments were conducted monthly.

### siRNA transfection and stable cell line generation

For transient knockout experiments, PC-3 cells were reverse transfected with 25 nM ON-TARGETplus Human SIX2 siRNA SMARTpool (siSIX2) (Dharmacon) and ON-TARGETplus Non-targeting Control siRNA (siCTRL) (Dharmacon) in Opti-MEM media (Gibco) using Lipofectamine RNAiMAX transfection reagent (Thermo Fisher Scientific). For NCI-H660 siRNA knockdown experiments, 50 nM siRNA was used. Transfections were performed in antibiotic-free maintenance media. NCI-H660 cells were incubated overnight with siRNA, followed by a 4-h recovery in complete medium before re-transfection for 4 h. In longitudinal experiments, cells were transfected again 7 days post-initial transfection.

For the generation of PC-3 cell line with nuclear labeling, cells were transduced with Incucyte Nuclight Red Lentivirus (Sartorius), resulting in stable nuclear expression of red fluorescent protein. To establish a stable cell line for fluorescence-based monitoring of cell cycle phases, PC-3 cells were transduced with IncuCyte Cell Cycle Green/Red Lentivirus Reagent (Sartorius) as described previously (18).

### SIX2 overexpression

The SIX2 overexpression and control lentiviral plasmids were done using Gateway cloning. For this SIX2 gene (clone ID 7508802; MGC Library; Genome Biology Unit supported by HiLIFE and the Faculty of Medicine, University of Helsinki, and Biocenter Finland) was PCR amplified using forward primer GGGGACAACTTTGTACAAAAAAGTTGGCACCATGTCCATGCTGCCCACCTTCGGCTTCA and reverse primer GGGGACAACTTTGTACAAGAAAGTTGGCAACTAGGAGCCCAGGTCCACGAGGTTGGCTG, and then cloned into Gateway donor vector pDONR221 to create entry clone SIX2-pENTR221. SIX2-pENTR221 was used to insert SIX2 gene into lentiviral vector pLenti7.3/V5-DEST, which includes Emerald Green Fluorescent Protein (EmGFP) for monitoring of protein expression. Control plasmid was created by inserting lacZ gene into pLenti7.3/V5-DEST. Both SIX2 OE and control lacZ constructs were generated by the Genome Biology Unit supported by HiLIFE and the Faculty of Medicine, University of Helsinki, and Biocenter Finland. Lentiviral vectors were produced by standard calcium phosphate transfections method at the National Virus Vector Laboratory (University of Eastern Finland). SIX2 overexpression and lacZ control vector were introduced to LNCaP cells using multiplicity of infection (MOI) of 5. SIX2 overexpression and control cells were sorted from non-transduced cells with FACS (CytoFLEX SRT) based on GFP fluorescence.

### Western blotting

Total proteins were extracted from adherent cells using ice-cold 1× TBS with protease inhibitors. Cells were suspended to 1× SDS containing complete protease inhibitors cocktail (Roche). Suspensions were sonicated 2 × 10 s 2 and μl 2-mercaptoethanol/100 μl 1× SDS was added and then incubated for 2 min at 95°C. To obtain nuclear and cytoplasmic fractions, NE-PER Nuclear and Cytoplasmic Extraction Reagent Kit (Thermo Scientific) and extraction according to protocol was used. 10 μl of protein sample was separated on 10% SDS-PAGE and transferred to a nitrocellulose membrane. Membranes were blocked in 5% non-fat dry milk in 1X Tris-buffered saline (TBS) with 0.1% Tween at room temperature for 1 h and incubated overnight at 4°C with primary antibodies at noted dilutions. The following day, membranes were washed in TBS with 0.1% Tween-20 for 15 + 5 + 5 min and incubated 1 hour at RT with secondary antibodies. Membranes were washed again in TBS with 0.1% Tween for 3 × 10 min and 10 min with TBS. Proteins were detected using Pierce^TM^ ECL Western Blotting Substrate kit (Thermo Scinentific) and chemiluminescent detection with ChemiDoc MP Imaging System (Bio-Rad). GAPDH was used as the internal reference protein to quantify protein levels using Image Lab Software (Bio-Rad). The antibodies and dilutions used for immunoblotting are shown in Supplementary Table S1.

### Real-time qPCR

To assess mRNA levels of genes in samples, RT-qPCR was performed. Total RNA was extracted from cells using TriPure Isolation reagent (Roche). 1 μg of total RNA was reversed transcribed using Transcriptor First Strand cDNA Synthesis Kit (Roche). Three biological replicates were analysed using LightCycler 480 SYBR Green I Master reagent (Roche) and the qPCR run was performed using LightCycler 480 Instrument II (Roche), two technical replicates per sample. The relative gene expressions were quantitated by using the 2^−ΔΔCt^ method (19). *GAPDH* was used to normalize amount of mRNA between samples. Primer sequences are shown in the Supplementary Table S2.

### RNA-seq

RNA used for RNA-sequencing (RNA-seq) libraries were quality controlled with Agilent 2100 Bioanalyzer using RNA 6000 Nano kit (Agilent, #5067-1511). Library preparation was performed by Novogene (Cambridge, United Kingdom). After the QC procedures, mRNA was enriched using oligo (dT) beads, and fragmented randomly. cDNA was synthesized by using mRNA template and random hexamers primer, after which a custom second-strand synthesis buffer (Illumina), dNTPs, RNase H and DNA polymerase I were added to initiate the second-strand synthesis. After a series of terminal repair and sequencing adaptor ligation, the double-stranded cDNA library was completed through size selection and PCR enrichment. Library quality was assessed with Agilent 2100 Bioanalyzer using DNA 1000 Analysis kit (Agilent, #5067-1504). Sequencing was done by Novogene (Cambridge, United Kingdom) using Illumina NovaSeq 6000 platform. Three biological replicate samples were sequenced. RNA-seq data was aligned to hg38 genome using STAR2.7 (20) with default settings and max 10 mismatches and max 10 multi-mapped reads. Differentially expressed genes were then analyzed with DESeq2 (21) through HOMER (22) for all comparisons.

### GSEA

Gene-Set Enrichment Analysis (GSEA) of differentially expressed genes was performed using GSEA software (v.4.3.2) from the Broad Institute (Massachusetts Institute of Technology) and Molecular Signatures Database (MSigDB) (v.2023.1) was used as reference for the results. The tool was run in classic mode to identify significantly enriched pathways. Pathways enriched with a nominal *P*-value <0.05 and false discovery rate (FDR) <0.25 were considered to be significant. All significantly differentially expressed genes (adjusted *P*-value < 0.05) from RNA-seq data analysis were used for analysis.

### ChIP-seq

For chromatin immunoprecipitation (ChIP), PC-3 cells were seeded onto 10 cm plates, 2 million cells per plate. Chromatin was fragmented to an average size of 150–300 bp by sonication (Diagenode, #UCD-300). SIX2 antibodies (10 μg per IP) were coupled to magnetic protein G Dynabeads (Invitrogen, 10004D) for at least 16 h, and sonicated lysates were incubated with antibody-coupled beads for o/n at 4°C. Sequencing libraries were generated using NEBNext Ultra II DNA Library Prep Kit (New England BioLabs, #E7645L) according to manufacturer's protocol. Analysis of library quality was done with Agilent 2100 Bioanalyzer using DNA 1000 Analysis kit (Agilent, #5067-1504). Two biological replicate samples were sequenced with Illumina NextSeq 500 (75SE).

### ATAC-seq

For assay for transposase-accessible chromatin with sequencing (ATAC-seq), LNCaP cells were seeded onto 10 cm plates, 2 million cells per plate. For nuclei isolation, the cell pellets were resuspended in a concentration of 3 million cells per ml in Buffer A [15 mM Tris–HCl (pH 8), 15 mM NaCl, 60 mM KCl, 1 mM EDTA, 0.5 mM EGTA, 0.5 mM spermidine (Sigma-Aldrich, S2626), 1× protease inhibitor cocktail]. Subsequently, equal volume of Buffer A with 0.04% (w/v) IGEPAL (Sigma, #I8896) was added, to obtain a concentration of 1.5 million cells per ml with 0.02% (w/v) IGEPAL. Samples were incubated on ice for 10 min, washed once with Buffer A without IGEPAL, and two times with ATAC resuspension buffer (10 mM NaCl, 10 mM Tris–HCl, 3 mM MgCl_2_). Isolation of nuclei was verified by Trypan Blue counting. Subsequently, 100 000 nuclei were subjected to Tn5 transposition reaction using 2.5 μl TDE1 from Nextera DNA Library Prep Kit (Illumina, #FC-121-1030). After adding the transposition reaction mix, the samples were incubated 45 min at 37°C with 800 rpm shaking, and subsequently DNA was purified using Monarch PCR & DNA Cleanup Kit (New England BioLabs, #T1030). DNA fragments were amplified with PCR and samples were barcoded using published primers (23). Amplified fragments were size selected (150–800 bp) using SPRIselect beads (Beckman Coulter, #B23318). Analysis of library quality was done with Agilent 2100 Bioanalyzer using High Sensitivity DNA Analysis kit (Agilent, #5067-4626). Two biological replicate samples were sequenced with Illumina NextSeq 500 (40PE).

### ChIP-seq and ATAC-seq data analysis

Read quality filtering and alignment to hg38 genome was performed using Bowtie2 (24) using default settings. Downstream data analysis was performed using HOMER. SIX2 peaks were called using default parameters on findPeaks with style factor, FDR <0.01, >25 tags, >4-fold over control sample and local background. ChIP input sample was used as a control. ATAC-seq peaks were called with findPeaks with style factor, FDR <0.01 and >6-fold over local background. Differential ATAC-seq peaks were defined with getDifferentialPeaks.pl (Poisson *P*-value < 0.0001, FC > 3) between specified treatments (unchanged, UN; increased, UP; decrease, DN). Aggregate plots and heatmaps were generated with 10 or 20 bp bins surrounding ±1 kb area around the center of the peak. All plots were normalized to 10 million mapped reads and further to local tag density, tags per bp per site, whereas box plots represented log_2_ tag counts. Statistical significance in the box plots was determined using unpaired two-sample t-test. Box plots were generated using Tukey method. Enrichment of sites to genomic elements was performed with annotatePeaks.pl. De novo motif searches were performed using findMotifsGenome.pl with the following parameters: 200 bp peak size window, strings with 2 mismatches, binomial distribution to score motif *P*-values, and 50 000 background regions. Gene-to-peak association was performed with annotatePeaks.pl measuring linear distance from target gene TSS to center of the peak. Distances were shown as cumulative distribution function from 0 to 100 kb with 10 kb intervals. Statistical significance was calculated with Kolmogorov–Smirnov test comparing differentially expressed genes (UP and DN) to expressed genes (UN). Pearson correlation coefficiency (PCC) between SIX2 binding sites and publicly available sequencing data, was done from normalized tag counts.

### Proliferation and apoptosis assays

To determine the proliferation rate, PC-3 cells stably expressing NucLight Red were seeded at the density of 7500 cells/well on a 96-well cell culture plate wells. For the proliferation and apoptosis assays in SIX2 overexpressing cells, LNCaP SIX2 OE and CTRL cells were seeded on a 96-well plate at a density of 5000 cells/well. One day after seeding, Enzalutamide (10 μM) or DMSO as control was added. The detection of apoptotic cells was carried out using the IncuCyte Caspase-3/7 red Dye for Apoptosis (Sartorius). Cell confluency and cell counts were imaged using IncuCyte S3 system (Sartorius) standard image scanning and calculated using the IncuCyte Basic Analyzer module (Version 2021C) and red fluorescence. For the apoptosis assay for PC-3 and NCI-H660 cells was carried out similar way but using the IncuCyte Caspase-3/7 green Dye for Apoptosis (Sartorius). A minimum of five technical replicates and two biological replicates were performed.

### Colony formation assay

Single cell suspension of cells stably expressing NucLight Red was plated in a 96-well cell culture plate, approximately 20 cells per well. Cells were reverse transfected with non-targeting control or SIX2 siRNA and re-transfected 5 days post initial transfection. Whole-well imaging with Incucyte Dilution Cloning scanning was used to track colony formation every 24 h over 9 days. A colony is defined to consist of at least 50 cells. Total cell count per colony was assessed using the IncuCyte Basic Analyzer module and counting of red fluorescent objects. 10 technical replicates and 2 biological replicates were performed.

### Cell migration Scratch Wound assay

To study the effects of SIX2 silencing to migration of PC-3 cells, scratch wound migration assay was performed and detected using the IncyCyte S3 live-cell imaging system. PC-3 cells were plated in IncuCyte ImageLock 96-well plates and reverse transfected with siRNAs. Three days after transfection at near 100% confluency the 96-well WoundMaker Tool (Sartorius) was used to create the wound. The wound was imaged every 2 h for a total of 12 h using Scratch Wound scan type in IncuCyte S3 Live-Cell Analysis System. IncuCyte Scratch Wound Analysis module was used to analyse cell movement and Relative Wound Density % values were used to estimate cell migration.

### Cell cycle assay

For cell cycle analysis, PC-3 cells stably expressing IncuCyte Cell Cycle Green/Red nuclear label were seeded in a 96-well plate wells. Fluorescence was monitored using the IncuCyte red and green fluorescence imaging and the images were analyzed using IncuCyte Basic Analyzer module. A minimum of five technical replicates and two biological replicates were performed.

### Spheroid assays

For spheroid assays, cells were seeded at the density of 2500 cells/well in a round bottom ultra-low attachment (ULA) plate and centrifuged 125 g, 10 min at RT. To analyse invasiveness of cells, 3 days after seeding, Cultrex Basement Membrane Extract (Bio-Techne) was added in the wells for a final concentration of 50%. To polymerize Cultrex, plate was incubated for 30 min at 37°C, and post polymerization complete culture media was added into the wells. Spheroid formation and growth was monitored using IncuCyte single spheroid scanning. Spheroid size and/or area of invasive cell area were measured using brightfield images and the IncuCyte Spheroid Analysis module.

### Immunofluorescence (IF)

For immunofluorescent staining, PC-3 cells were plated on coverslips, fixed using 4% paraformaldehyde for 20 min, washed 3× in PBS followed by permeabilization with 0.1% Triton-X with 1% bovine serum albumin (BSA) for 15 min. Cells were washed 3× in PBs, incubated with primary antibody (β-catenin, Santa Cruz) at 1:100 concentration for at 4°C overnight in humidity chamber. The following day, cells were washed 3× in PBS and incubated with secondary antibody, Goat anti-Mouse IgG (H + L) Alexa fluor 633 (Invitrogen, #A21052) 1:500, for 1 h at room temperature, followed by incubation with DAPI (Thermo) 1 μg/ml for 10 min at room temperature. Cells were mounted on slides using ProLong Diamond Antifade Mountant (Thermo). Fluorescent images were taken using 40× oil immersion objective using ZEISS LSM 900 with airyscan confocal microscope and ZEISS blue software. The measurement of fluorescence intensity of nuclear β-catenin in siCTRL and siSIX2 PC-3 cells was performed on confocal images. The fluorescence intensity measurement was executed using the ImageJ software (Ver. 1.53v) and BioVoxxel Toolbox (Ver. 2.6.0).

### Zebrafish *in vivo* metastasis and dissemination assays

Zebrafish experiments were carried out under the license ESAVI/31414/2020 (granted by Project Authorization Board of Regional State Administrative Agency for Southern Finland) according to the regulations of the Finnish Act on Animal Experimentation (62/2006) in the Zebrafish Core Facility of Turku Bioscience Centre, University of Turku and Åbo Akademi University, Turku, Finland, supported by Biocenter Finland). The study was carried out in compliance with the ARRIVE guidelines. The embryo xenograft procedure is described in detail in (25,26). Pigment-deficient casper embryos were obtained by natural spawning. The embryos were kept in E3 + PTU until transplanted with CellTracker Green CMFDA labeled PC-3 cells at 2 days post-fertilization using Nanoject II microinjector (Drummond Scientific). Approximately 400–500 cells were transplanted into yolk sac of the embryos, and after transplantation the embryos were kept at 33°C incubator. At 1-day post injection (dpi) the successfully transplanted embryos were selected under Zeiss AxioZoom fluorescence stereomicroscope and transferred to 96-well glass bottom plate (1embryo/well). Embryos were immobilized using 200 mg/l tricaine prior imaging and imaged at 1 and 4 dpi time points using Nikon Eclipse Ti2-E microscope, 2× NA 0.06 objective. Fluorescence images were captured using Lumencor Spectra X LED 475/28 nm illumination, Chroma 8400v2 filter and Hamamatsu sCMOS Orca Flash4.0 camera. Brightfield images were captured with unfiltered white light illumination from a halogen lamp.

In the image analyses, ImageJ/FIJI software was used. The number of invaded cells were counted manually based on fluorescence. Only invading cells outside the yolk sac were counted. Invading cells in the lens were not counted as the lens tend to have prominent autofluorescence. Embryos having significant malformations were excluded from the analysis. Samples were blinded for analyses.

For the zebrafish in vivo dissemination assay, the siCTRL and siSIX2 cells were injected in zebrafish vein and the invaded cells were measured after 24 h similarly as before (27). Briefly, PC-3 cells were washed with PBS, stained with CellTracker Green CMFDA dye (5 μm, Thermo Fisher Scientific) and detacher and detached with trypsin–EDTA in a single incubation step at 37 °C. Subsequently, cells were pelleted by centrifugation and washed with PBS twice. This was followed by filtration through 40 μm mesh into Falcon FACS tube (Corning, Corning, NY, USA, 352235) and pelleting cells by centrifugation. Finally, cells were resuspended into 30 μL of injection buffer (2% PVP in PBS) and kept on ice until transplanted. The embryos were obtained through natural spawning, and were cultured in E3-medium (5 mm NaCl, 0.17 mm KCl, 0.33 mm CaCl_2_, 0.33 mm MgSO_4_) at 33 °C. At 2 or 3 days post-fertilization, the embryos were anesthesized with 200 mg ml^−1^ Tricaine and embedded in 0.7% low-melting point agarose. Subsequently, the siCTRL and siSIX2 cell suspensions were microinjected into common cardinal vein (duct of Cuvier) of the embryo using glass capillaries (Transfertip), CellTramVario microinjector and InjectMan micromanipulator (all from Eppendorf, Hamburg, Germany). Embryos were liberated from the agarose gel using forceps and successfully transplanted embryos were selected to the experiment. After overnight incubation at 33°C, the embryos were anesthesized again with Tricaine and imaged using Zeiss StereoLumar V12 fluorescence stereomicroscope. The number of surviving cells was counted manually from the images using ImageJ.

### Statistical analyses

All statistical analyses and visualization were performed using GraphPad Prism (version 9), unless otherwise specified. Representative data such as western blot and immunofluorescence staining has been repeated at least twice with independent biological samples and technical replicates. Representative data shown for *in vitro* experiments have been repeated at least twice. For *in vitro* assays, unpaired, two-tailed, Student's *t*-tests were performed to analyse statistical significance between groups in bar graphs, box and whisker and longitudinal experiments. For longitudinal experiments statistical analysis was performed at the final time point. *In vitro* assays *P*-value <0.05 was considered to be significant. Significances are indicated as follows in the figures: * *P*< 0.05; **P< 0.01; *** *P*< 0.001 and **** *P*< 0.0001.

## Results

### Androgen receptor inhibition opens chromatin regions of SIX family protein motifs

**Figure 1.**
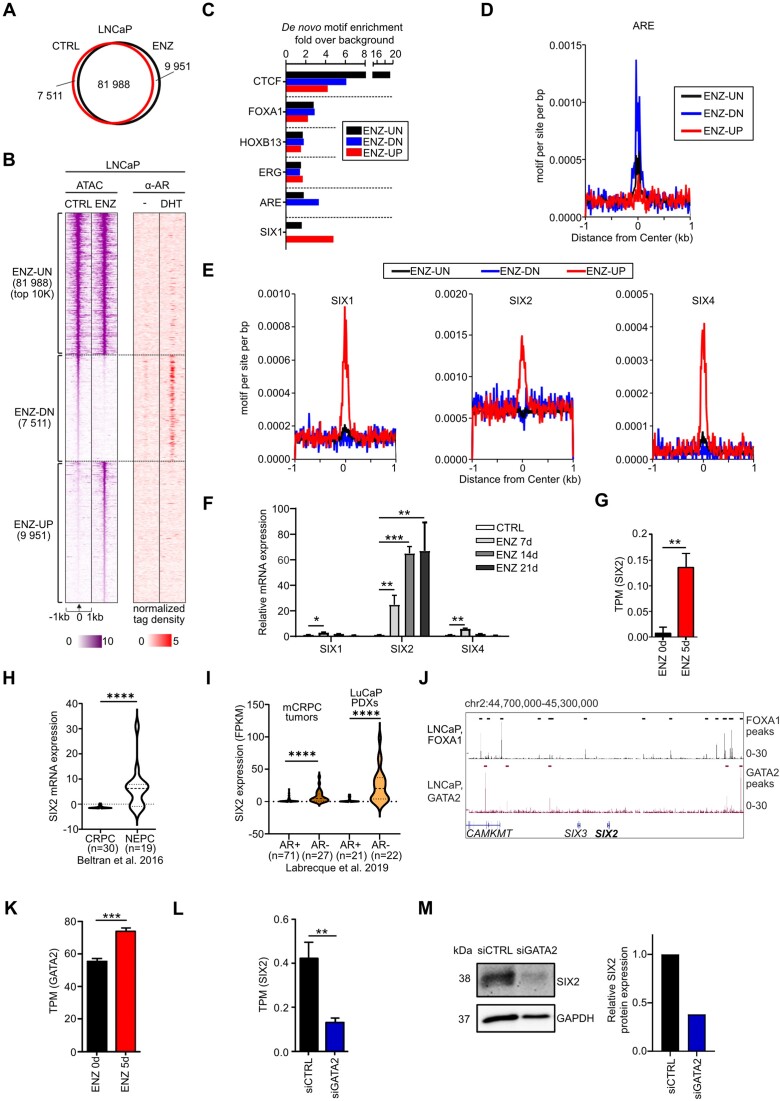
Androgen receptor inhibition opens chromatin in motifs of SIX proteins. (**A**) Venn diagram showing 9951 opened and 7522 closed regions by 3-week ENZ in LNCaP cells. (**B**) Heatmaps displaying assay for transposase-accessible chromatin with sequencing (ATAC-seq) data at chromatin regions unchanged (ENZ-UN), opened (ENZ-UP) or closed (ENZ-DN) by 3-week ENZ in LNCaP cells (left) and AR ChIP-seq with or without AR activating dihydrotestosterone (DHT) (right). (**C**) De novo motif analysis of ENZ unchanged (ENZ-UN), opened (ENZ-UP) or closed (ENZ-DN) chromatin sites. (**D**) AR motif is enriched in chromatin sites closed by ENZ. (**E**) Motifs of three SIX family proteins, SIX1, SIX2 and SIX4 are enriched in chromatin sites opened by ENZ. (**F**) Bar graph depicting relative mRNA expression of SIX1, SIX2 and SIX4 in LNCaP cells exposed to ENZ for 1–3 weeks. (**G**) SIX2 expression compared to control (ENZ0) LNCaP cells, analysed using publicly available RNA-seq data from LNCaP cells (28). (**H**) SIX2 mRNA expression in AR positive CRPC and AR negative NEPC patient cohorts (10). Significance assessed using a two-tailed unpaired *t*-test. (CRPC *n* = 30 and NEPC *n* = 19). (**I**) SIX2 expression in AR positive and AR negative mCRPC tumors and LuCaP PDXs (11). Significance assessed using a two-tailed unpaired *t*-test (mCRPC tumors AR+ *n* = 71 and AR– *n* = 27; LuCaP PDXs AR+ *n* = 21 and AR– *n* = 22). (**J**) Visualization of binding sites of GATA2 and FOXA1, the closest transcription factor binding sites in the SIX2 gene locus. (**K**) ENZ exposure for 5 days (ENZ5d) increases the mRNA expression of GATA2 compared to control (ENZ0) LNCaP cells, analysed using publicly available RNA-seq data from LNCaP cells (28). (**L**) Silencing of GATA2 using siRNA (siGATA2) downregulates SIX2 mRNA in the LNCaP cells analyzed using publicly available data (28). (**M**) Silencing of GATA2 downregulates the protein expression of SIX2 in PC-3 cells assessed using western blot (left). The quantification of the SIX2 expression in siCTRL and siGATA samples in comparison to GAPDH loading control was calculated and shown (right).

Reprogramming of prostate cancer cells post androgen receptor inhibitor treatment is accompanied by extensive transcriptional re-wiring (10,11). To study the lineage plasticity post ENZ and identify master transcription factors responsible for reprogramming cellular fate decisions in treatment-resistant prostate cancer, we utilized transposase-accessible chromatin sequencing (ATAC-seq) after a long-term (3 weeks) ENZ exposure in AR-positive LNCaP cells. Our data revealed that while the majority of open chromatin sites remained insensitive to ENZ (ENZ-UN), ENZ treatment led to the emergence of multiple open (ENZ-UP) and closed (ENZ-DN) chromatin regions. Example genome browser tracks are provided in Supplementary Figure S1a. Surprisingly, there were more novel accessible regions opened by ENZ (9951 regions) than closed (7511 regions) (Figure 1A, B). To determine enriched motifs in each group, we conducted *de novo* motif analysis on ENZ-UN, ENZ-DN and ENZ-UP sites. As expected, the androgen response element (ARE) motif and AR chromatin binding sites were enriched at ENZ-DN sites (Figure 1B–D). Interestingly, when motifs were ranked by *P*-value, we discovered that the DNA binding motif for the homeodomain transcription factor Sine Oculis Homeobox 1 (SIX1) exhibited the highest enrichment among accessible chromatin regions (ENZ-UP) post ENZ treatment (Figure 1C).

Given the similarity in binding motifs within the SIX-family of transcription factors, we conducted a more detailed investigation into the motif enrichment of SIX1, SIX2 and SIX4 (Supplementary Figure S1b). Our analysis revealed that in addition to SIX1, both SIX2 and SIX4 transcription factor motifs were specifically enriched in the ENZ-UP regions but not in the ENZ-DN or ENZ-UN regions (Figure 1E), suggesting the induced activity of SIX proteins in response to ENZ. To further understand the impact of ENZ on SIX genes, we examined the mRNA expression of SIX1, SIX2 and SIX4 in response to ENZ in LNCaP cells using RT-qPCR (Figure 1F). SIX2 mRNA expression showed increases of 30-, 70- and 75-fold after 7, 14 and 21 days of ENZ exposure, respectively, while only minor increases were observed in SIX1 and SIX4 expressions. Complementarily analysis of publicly available RNA-seq data (28) indicated a significant increase in SIX2 expression after 5-day ENZ (ENZ5d) in LNCaP cells (Figure 1G). Consistent with these findings, an increase in SIX2 protein was observed in LNCaP cells exposed to ENZ (Supplementary Figure S1c). Taken together, our results demonstrate that ENZ induces the opening of chromatin regions containing the SIX2 motif, leading to a substantial increase in SIX2 expression. These findings suggest a potential role for SIX2 in establishing treatment-induced lineage plasticity in prostate cancer.

### SIX2 is highly expressed in neuroendocrine and AR negative prostate cancer tumors and correlates with SOX2, CGA, NSE and SYP

To assess the expression of SIX2 in prostate cancer tumors, we examined SIX2 levels in patient and patient-derived xenograft (PDX) tumors (10,11). Interestingly, our results revealed significantly higher SIX2 mRNA levels in neuroendocrine prostate cancer (NEPC) carcinoma tumors compared to CRPC tumors (Figure 1H). Additionally, SIX2 expression was elevated in patient tumors and PDXs with low AR activity compared to adenocarcinoma tumors exhibiting high AR activity (Figure 1I). Analysis of patient tumor data (10) revealed a negative correlation between SIX2 mRNA expression and AR and *KLK3* (encoding PSA). Conversely, SIX2 showed positive correlations with SOX2, CGA, ENO2 (NSE) and SYP, known features of NEPC (Supplementary Figure S1d). In summary, these results indicate that SIX2 is highly expressed in NEPC and AR-negative tumors, and correlates with neuroendocrine features.

### SIX2 expression is regulated by Enzalutamide-induced GATA2

Our data show that ENZ opens chromatin regions containing the SIX2 motif and induces SIX2 expression. In addition, we observed a negative correlation between SIX2 expression and AR/PSA in patient tumor data. While we initially hypothesized that AR might control SIX2 expression by binding to its chromatin site, we could not detect AR binding at the SIX2 locus in publicly available ChIP-seq data (29–31). Since SIX2 expression significantly increases after several days of ENZ exposure (Figure 1F), it is likely regulated though secondary effects following AR suppression. Interestingly, we found that the closest transcription factor binding sites in the SIX2 gene locus are for GATA2 and FOXA1 (Figure 1J). This led to hypothesize that ENZ-induced GATA2, with binding sites distinct from AREs (30), could explain the ENZ-induced regulation of SIX2 over time. Analyzing ENZ-exposed RNA-seq data (28), we observed a significant increase in GATA2 mRNA in ENZ5d-exposed cells (Figure 1K). To investigate whether SIX2 expression is regulated by GATA2, we analyzed data from GATA2-silenced LNCaP cells (32). The results indicated that siGATA2 significantly downregulates SIX2 mRNA expression (Figure 1L). In addition, we observed a downregulating effect on SIX2 protein in GATA2-silenced PC-3 cells (Figure 1M). In conclusion, our results suggest that ENZ-induced SIX2 could be regulated via GATA2, supported by the finding that GATA2 and FOXA1 are the closest transcription factor binding sites in the SIX2 gene locus. However, a detailed investigation into the molecular mechanism underlying how SIX2 becomes upregulated over time under AR suppression is warranted.

### SIX2 regulates stem cell-like plasticity in AR-negative prostate cancer cells via regulation of Wnt ligand expression and reduction of nuclear β-catenin

**Figure 2.**
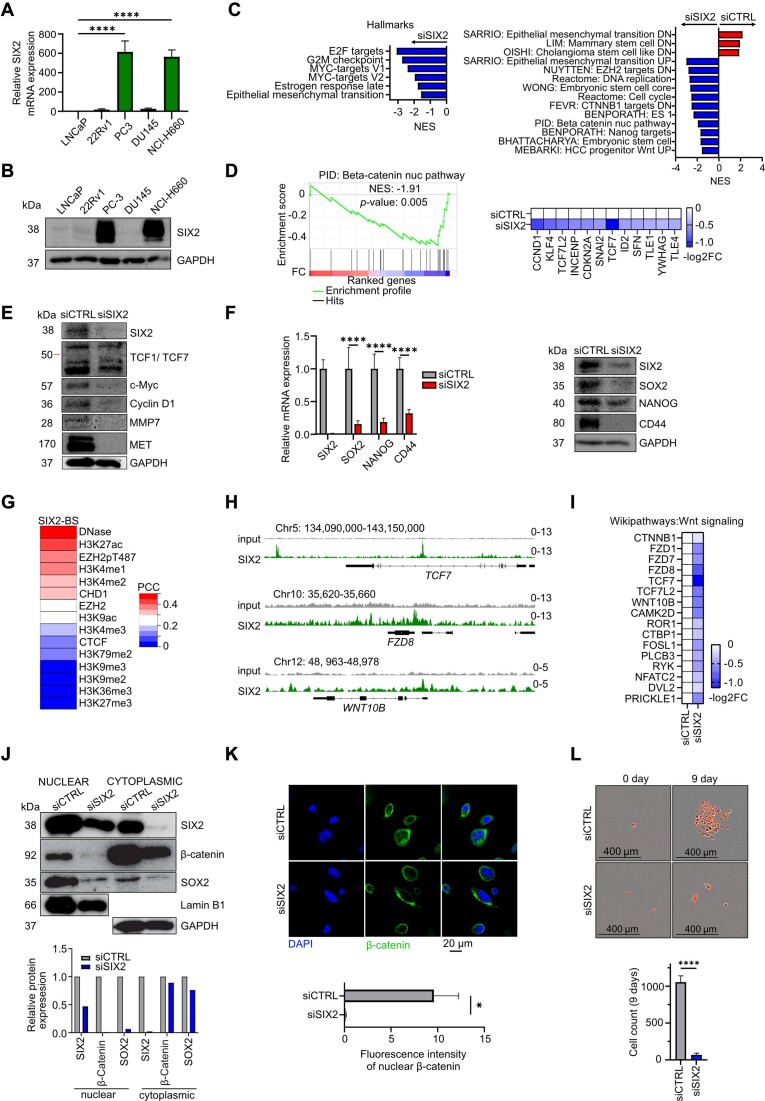
SIX2 depletion reduces cell stemeness via downregulation of Wnt/β-catenin signalling and β-catenin nuclear translocation. (**A**) *SIX2* mRNA expression in PCa cell lines normalised to GAPDH (*n* = 3). (**B**) Western blot shows SIX2 protein expression in PCa cell lines, GAPDH was used as loading control. (**C**) GSEA of Hallmark gene sets in the SIX2 silenced PC-3 cells reveal negatively enriched gene sets related in cell proliferation and epithelial mesenchymal transition. Curative gene sets enriched in the SIX2 silenced PC-3 cells reveal positive and negative enrichment of gene sets indicating reduced stemness, epithelial mesenchymal transition (EMT) and Wnt signaling. (**D**) GSEA enrichement plot (left) and heatmap (right) for ‘β-catenin nuc pathway’ in SIX2 silenced PC-3 cells compared to control. (**E**) Western blot shows protein expression of Wnt/β-Catenin activated targets in siCTRL and siSIX2 PC-3 cells. (**F**) SIX2, SOX2, NANOG and CD44 mRNA expression normalised to *GAPDH* expression in SIX2 depleted PC-3 cells compared to control (*n* = 3) (left) and protein expression of SIX2, SOX2, NANOG and CD44, GAPDH used as loading control (right). (**G**) PCC plot showing SIX2 binding site correlation with published DNase-seq and ChIP-seq data. (**H**) Visualization of genomic loci of *TCF7, FZD8* and *WNT10b* using IGV showing SIX2 occupancy. (**I**) Heatmap repressenting genes of Wikipathways:Wnt siganlign gene set that have SIX2 binding site closer than 10 kb of TSS and whose expression is downregulated after SIX2 silencing. (**J**) Western blot of SIX2, β-catenin and SOX2 in control and SIX2 silenced PC-3 cell nuclear and cytoplasmic protein fractions, LAMIN B1 and GAPDH used as loading controls (top) and the quantification of the protein expression in siCTRL and siSIX2 samples in comparison to loading controls was calculated and shown (bottom). (**K**) Visualization of nuclear localization of β-catenin in control versus cytoplasmic localization in SIX2 silenced cells, ‘ β-catenin’ (green), ‘DAPI’ (blue), scale bar = 20 μm (top) and nuclear β-catenin fluorescence intensity quantification (*n* = 5 per group). **l** Microscopy images of colony formation assay taken by IncuCyte on days 1 and 9 (top) and bar graph obtained by calculating the count of cells from control and SIX2 silenced PC-3 cells with red nuclear stain (bottom) (*n* = 10).

Our data revealed that AR inhibition by ENZ opens the chromatin area of SIX2 and SIX2 is highly expressed in AR-negative patient tumors. As SIX2 plays a role in regulating stem cell phenotypes (13,14), we hypothesized that SIX2 is an early driver of stem cell-like plasticity in prostate cancer enabling development of treatment-resistant phenotypes. To explore this hypothesis, we first assessed SIX2 expression across different prostate cancer cell lines to identify suitable models for studying SIX2 and its functions in prostate cancer. The mRNA and protein levels of SIX2 were analyzed in prostate cancer cell lines and as expected the SIX2 mRNA expression was highest in androgen-negative PC-3 and NEPC NCI-H660 cells while AR positive LNCaP and 22Rv1 cells expressed very little of SIX2 (Figure 2A). Same phenomenon was observed on a protein level using immunoblotting analysis (Figure 2B). Notably, AR-negative DU145 cells which though have lower metastatic potential compared to PC-3, did not display elevated SIX2 expression (Figure 2A and B). Finally, analysis of publicly available chromatin accessibility data showed that the SIX2 promoter is accessible in PC-3 and NCI-H660 cells, similar to the increased promoter accessibility observed in LNCaP cells upon ENZ treatment (Supplementary Figure S2a). Based on these results, we selected two high SIX2-expressing cell lines, PC-3 and NCI-H660, which are frequently used as suitable models for studying NEPC and AR-negative phenotypes, for further functional studies.

Next, to evaluate the impact of SIX2 silencing in high SIX2-expressing PC-3 cells on lineage plastic states, we performed RNA-seq and gene set enrichment analysis (GSEA) on SIX2 silenced PC-3 cells. SIX2 silencing was done using SIX2 targeted siRNA (siSIX2) transfections, with non-target siRNA (siCTRL) used as a control. In addition, to clarify how SIX2 carries out its effects, we mapped the genome-wide occupancy and chromatin binding sites of SIX2 using chromatin immunoprecipitation sequencing (ChIP-seq) in PC-3 cells.

The GSEA of Hallmark and curative gene sets revealed that SIX2 silencing downregulates cell cycle, epithelial–mesenchymal transition and stemness signatures, including embryonic stem cell genes and NANOG targets (Figure 2C). In addition, GSEA revealed negative enrichment in the gene set PID: Beta-catenin nuc pathway, encompassing genes related to the regulation of nuclear β-catenin signalling and target gene transcription (normalised enrichment score (NES) –1.91, *P*-value of 0.005) (Figure 2D). Other β-catenin and Wnt-related gene sets also exhibited negative enrichment upon SIX2 silencing (Figure 2C). The RT-qPCR results validated the RNA-seq findings, consistently demonstrating the downregulation of INCENP, CCND1, TCF7 and FZD8 in SIX2-silenced PC-3 cells in both RNA-sequencing and RT-qPCR analyses (Supplementary Figure S2b). Moreover, protein expressions of TCF7 and canonical β-catenin targets c-Myc, Cyclin D1, MMP7 and MET were decreased (Figure 2E), suggesting that SIX2 regulates nuclear β-catenin signalling and target gene transcription. Similar results were observed in NCI-H660 cells (Supplementary Figure S2c). These findings were further supported by SIX2 overexpression experiments, where increased protein levels of c-Myc, Cyclin D1, MMP7 and MET were observed in LNCaP cells (Supplementary Figure S2d). Furthermore, we confirmed that SIX2 silencing significantly suppressed the expression of SOX2 and NANOG, known treatment resistance-associated pluripotency markers (33), and CD44, a recognized cancer stem cell marker (34), in PC-3 cells based on RT-qPCR and western blot analyses (Figure 2F), indicating decreased cell stemness. Additionally, a reduced mRNA expression of SOX2 was observed in SIX2-silenced NCI-H660 cells (Supplementary Figure S2e).

The ChIP-seq analysis revealed nearly 50000 binding sites for SIX2 (Supplementary Figure S2f). Interestingly, these SIX2 binding sites occurred at active chromatin sites, showing enrichment of open chromatin, the active enhancer/promoter mark H3K27ac, and active enhancer marks H3K4me1 and H3K4me2 in PC-3 cells (Figure 2G, Supplementary Figure S2g). Genomic loci visualization from ChIP-seq further illustrated significant binding of SIX2 to several gene promoters associated with the TCF/LEF/β-catenin transcriptional complex, including *TCF7*, as well as promoters of Wnt ligands and receptor genes such as *FZD8* and *WNT10b* (Figure 2h, Supplementary Figure S2h), indicating direct regulation of their expression. Analysing the overlap of SIX2 peaks with differentially expressed genes (DEGs) from RNA-seq revealed that siSIX2 versus siCTRL DEGs are in closer proximity to SIX2 binding sites compared to genes unresponsive to siSIX2 (Supplementary Figure S2i). Notably, downregulated genes with SIX2 binding sites closer than 10 kb include Wnt signalling-associated genes (Figure 2I), further supporting the notion that SIX2 regulates cancer stemness through direct transcriptional regulation of Wnt pathway ligands and receptors. The combined results from RNA-seq and ChIP-seq suggest that SIX2 depletion leads to the reduced expression of Wnt pathway genes through direct regulation, potentially preventing β-catenin nuclear translocation and Wnt signalling-induced stemness and cell plasticity in AR-negative prostate cancer cells.

To test whether SIX2 influences Wnt signalling by modulating nuclear β-catenin, we assessed the nuclear and cytoplasmic compositions of β-catenin in response to SIX2 depletion. Western blot analysis was conducted in PC-3 and NCI-H660 cells, and fluorescence staining was performed in PC-3 cells. Concurrently, we determined the cellular localization of the pluripotency factor SOX2 in response to silenced SIX2 using western blot. The results revealed that silencing of SIX2 led to a reduction in nuclear β-catenin levels in PC-3 (Figure 2J, K), suggesting that SIX2 can mediate its effects by regulation of Wnt/β-catenin signalling. A modest reduction of nuclear β-catenin was also seen in NCI-H660 cells, where an increase in cytoplasmic β-catenin was also observed (Supplementary Figure S2j). In addition, nuclear SOX2 levels decreased in response to SIX2 silencing in both PC-3 and NCI-H660 cells.

In the end, we aimed to confirm the effect of SIX2 silencing on the stem-like potential of AR-negative prostate cancer cells. Clonogenic activity serves as a sensitive indicator of undifferentiated cancer stem cells (35). Since AR-negative PC-3 prostate cancer cells are known to establish colonies from single cells, we explored whether SIX2 depletion could prevent the formation of PC-3 cell colonies. We utilized PC-3 cells stably expressing a red nuclear label, grew single cell colonies and monitored the cell colony formation over 9 days using IncuCyte S3 and fluorescence imaging. The results revealed that SIX2 silencing completely inhibits the ability of a single PC-3 cell to grow into a colony whereas single siCTRL control cells were capable of forming colonies in the culture (Figure 2K, Supplementary Figure S2k). These results indicate a reduced cell stemness in SIX2-silenced cells.

In summary, our results show that SIX2 depletion reduces stem cell-like plasticity via the direct regulation of Wnt ligand expression, nuclear β-catenin, and the colony formation capacity in AR-negative prostate cancer cells.

### SIX2 depletion induces MET and inhibits cell proliferation

**Figure 3.**
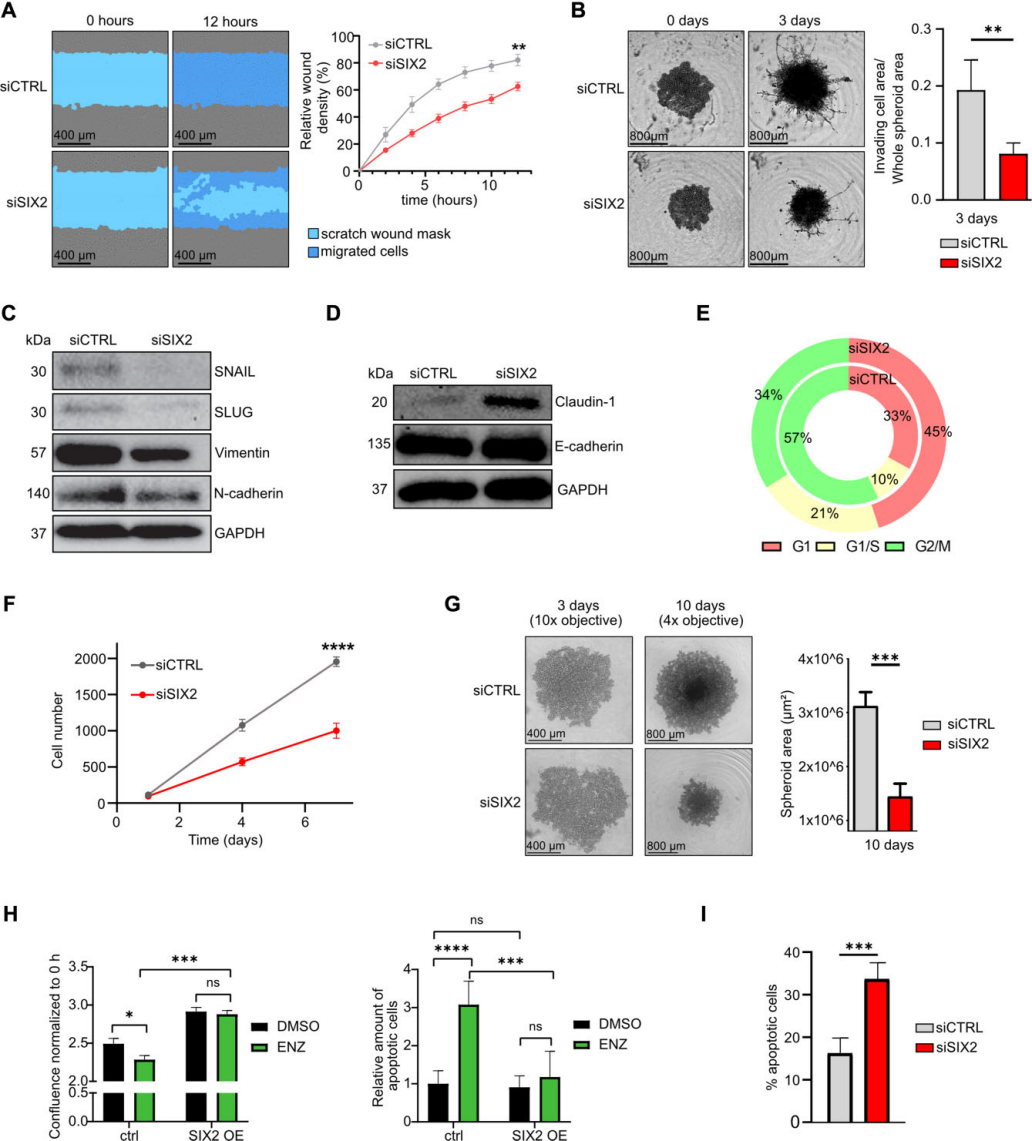
SIX2 is a potent regulator of EMT, migration and invasion. (**A**) Images from scratch wound assay of siCTRL and siSIX2 transfected PC-3 cells at 0 and 12 h post wound scratching (left). The scratch wound mask shown as cyan and migrated cells as blue. Relative wound density, ratio of the occupied area of the initially scratched area to the total area of the scratch, as a function of time (right). Error bars indicate standard deviation about the mean (*n* = 6). (**B**) Microscopy images showing the spheroids 0 and 3 days after adding Cultrex (left) and graph showing invading cell areas (μm²) in siCTRL versus siSIX2 transfected PC-3 cells (right). Reported as mean ± SD (*n* = 5). (**C**) Western Blot shows protein expressions of EMT markers in siCTRL and siSIX2 transfected PC-3 cells. (**D**) Expressions of epithelial marker proteins Claudin-1 and E-cadherin in siCTRL and siSIX2 transfected PC-3 cells assessed using western blot. (**E**) Cell cycle phases comparing PC-3 siSIX2 versus siCTRL reported as mean in percentage. *P-*value of two-tailed unpaired t-test G0-G1 = 0.0005, G1/S < 0.0001, G2 = 0.004 (*n* = 6). (**F**) Proliferation of PC-3 cells following siRNA transfections reported as mean ± SD (*n* = 6). (**G**) Representative images of PC-3 spheroids imaged on 3- and 10-days post siRNA transfections (left) and spheroid size reported as μm² 10-days post siRNA transfections in siCTRL versus siSIX2 transfected PC-3 cells reported as mean ± SD (*n* = 5) (right). (**H**) Bar graphs representing confluence (left) and apoptotic cells (right) of DMSO and ENZ exposed CTRL and SIX2 OE cells (*n* = 8). (**I**) Bar graph representing siCTRL and siSIX2 PC-3 cell apoptosis reported as percentage of apoptotic cells, mean ± SD (*n* = 5).

We showed that SIX2 silencing prevented Wnt signalling by decreasing the expression of Wnt pathway-related genes and nuclear β-catenin, leading to impaired colony formation in PC-3 cells. As Wnt signalling is also a key regulator of epithelial mesenchymal transition (EMT), migration, invasion, and proliferation, we further evaluated the effects of SIX2 depletion on these cellular phenotypes in PC-3 cells. The GSEA indicated a reduced EMT in the RNA-seq data of SIX2-silenced cells (Figure 2C). This suggests that SIX2 silencing induces as shift from mesenchymal-like cells back to epithelial cells, a process known as mesenchymal-epithelial transition (MET). To assess the impact of SIX2 depletion on metastatic properties, we conducted a scratch wound assay using IncuCyte, demonstrating that silencing SIX2 attenuated PC-3 cell migration (Figure 3B, Supplementary Figure S3). In addition, we investigated spheroid growth and invasion by growing SIX2-depleted PC-3 cells in spheroids surrounded by Cultrex Basement Membrane Extract. The spheroid growth and the invading of cells was monitored using IncuCyte S3. The results revealed that no invaded cells were observed in the surrounding basement membrane after SIX2 depletion, unlike in siCTRL-transfected control cells (Figure 3B). Finally, we explored whether SIX2 depletion induces MET in PC-3 cells by analysing the protein expressions of mesenchymal markers SNAIL, SLUG, Vimentin, and N-cadherin, as well as epithelial markers Claudin-1 and E-cadherin, using western blot. The results revealed that silencing SIX2 suppressed the protein expression of SNAIL and SLUG, known modulators of EMT and invasion (Figure 3C). Moreover, the expression of Vimentin and N-cadherin were suppressed (Figure 3C), while Claudin-1 was increased (Figure 3D), confirming a transition to an epithelial state in response to SIX2 silencing.

Analysis of the RNA-seq data revealed a downregulation of genes encoding regulators of cell cycle progression, including *CCND1 (*encoding Cyclin D1), in SIX2 depleted cells. This was further validated by a decrease in the protein expression of Cyclin D1 using immunoblotting (Figure 2E). Considering that *CCND1* is a key target gene of Wnt/β-catenin signalling (36), we evaluated the effects of SIX2 silencing on cell cycle regulation and cell proliferation. To monitor cell cycle phases, we generated PC-3 cells stably expressing fluorescence-tagged Cdt1 and Geminin, indicators of the cell cycle, using IncuCyte Cell Cycle lentivirus reagent. Upon SIX2 knockdown using siRNA, we observed a 12% increase in the G0-G1 population (*P*-value 0.0005) compared to the control (Figure 3E). Using PC-3 cells with a stable red nuclear label, we detected decreased cell proliferation capacity in both 2D culture and 3D spheroid culture (Figure 3F), as well as reduced spheroid growth (Figure 3G) in SIX2-silenced cells. The decrease in Snail protein level was also observed in PC-3 cells cultured in 3D spheroids (Supplementary Figure S4a). In addition, overexpression of SIX2 led to an increase in cell proliferation and a reduction in ENZ-induced apoptosis, indicating conferred ENZ resistance in SIX2-overexpressed cells (Figure 3H). Moreover, analysis of apoptosis using IncuCyte caspase-3/7 green dye revealed that SIX2 silencing induces caspase-3/7-mediated apoptosis in PC-3 cells (Figure 3I, Supplementary Figure S4b). Similar effects on cell apoptosis were observed in NCI-H660 cells (Supplementary Figure S4c).

Taken together, these results confirm that SIX2 silencing induces MET, reduces spheroid growth and invasion, inhibits cell migration, and halts cell cycle progression in prostate cancer cells.

### SIX2 depletion inhibits cell invasion *in vivo* in PC-3 cell zebrafish xenograft models

**Figure 4.**
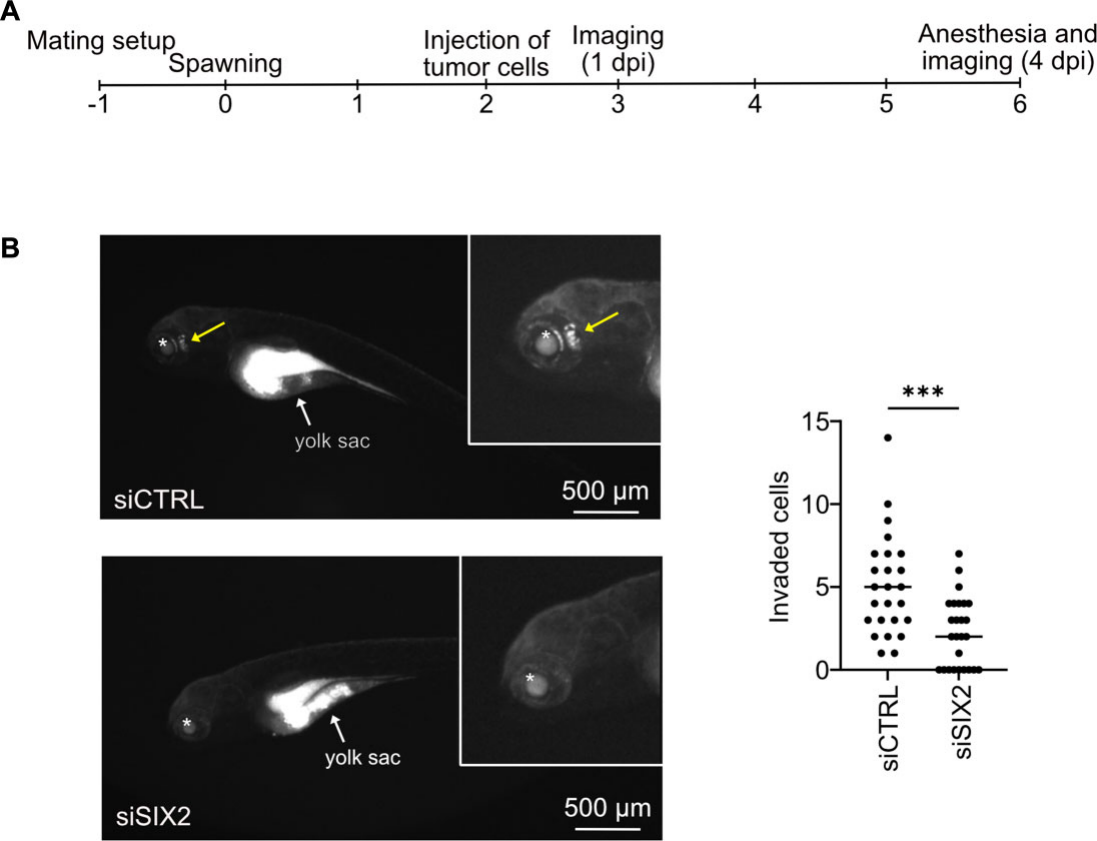
SIX2 depletion reduces invasion of PC-3 cells *in vivo*. (**A**) Timeline of zebrafish xenograft experiment. (**B**) Representative fluorescence microscopy images of zebrafish four days post injection (dpi) with siCTRL and siSIX2 transfected cells (left). Fluorescence in green fluorescence (CellTracker Green CMFDA) channel shown. Tumor cells invaded outside yolk sac are marked with an arrow and unspecific fluorescence in eye with an asterisk (*). Quantification of PC-3 cells invaded outside yolk sac at 4 dpi (siCTRL *n* = 23, siSIX2 *n* = 23) (right).

Our data revealed that SIX2 depletion reduces stemness and metastatic potential of AR negative prostate cancer cells *in vitro*. To assess the effect of SIX2 depletion on prostate cancer cell invasion *in vivo*, fluorescently stained SIX2-silenced and control PC-3 cells were microinjected into yolk sacs of zebrafish embryos. For a longitudinal analysis of cell invasion, the embryos were placed in 96-well plates. After 4 days, the ability of cells to invade and form distant colonies was evaluated using intravital fluorescence microscopy (Figure 4A). Fluorescence imaging and quantification revealed that while control cells invaded rapidly and formed distant tumors in zebrafish head and tail, silencing of SIX2 significantly decreased the number of invaded cells in zebrafish four days post injection (Figure 4B). These results underscore the role of SIX2 in mediating the invasion of PC-3 prostate cancer cells *in vivo*. Moreover, SIX2 silencing led to a decreased number of invading cells per embryo compared to siCTRL cells also in an additional zebrafish *in vivo* dissemination assay. In this assay, siCTRL and siSIX2 cells were injected into the zebrafish blood vein, and the number of invaded cells was measured after 24 h (Supplementary Figure S5).

## Discussion

Prostate cancer is a major global health concern, and second-generation AR pathway inhibitors such as ENZ or abiraterone are widely used as a first-line therapy for advanced prostate cancer (1,2). However, the development of resistance to AR inhibitors is a clinical challenge. Lineage plasticity, wherein prostate cancer cells switch their phenotype, is a contributor to therapy resistance and metastatic spread (3–5). Identifying novel regulators of cellular plasticity programs in prostate cancer cells is critical for developing effective therapies and overcoming resistance.

This study aimed to find novel transcriptional regulators associated with increased lineage plasticity induced by ENZ in prostate cancer cells. Through ATAC-seq analysis of ENZ-altered chromatin architecture, SIX2 emerged as one of the top enriched motifs in long-term ENZ-exposed LNCaP cells. Inhibition of the AR pathway was coupled with increased expression of SIX2. Furthermore, elevated levels of SIX2 were observed in AR-negative patient tumors compared to AR-positive tumors, suggesting its potential role as a candidate regulator of ENZ-induced plasticity and therapy resistance in prostate cancer. Since the increased SIX2 expression requires prolonged ENZ treatment, likely secondary and indirect events due to AR suppression causes the regulatory change in SIX2. Our data suggests that this regulation may be mediated via ENZ-induced GATA2 (32), whose binding site is close to the SIX2 gene locus. Notably, GATA2 depletion led to a significant downregulation of SIX2. However, the detailed molecular mechanism of SIX2 gene regulation under AR suppression requires further investigation.

SIX proteins are transcription factors that generally play a pivotal role in embryonic developmental processes (37). Building on the observation that SIX2 plays a central role maintaining cells in a stem cell stage during kidney development (12), mediates cell stemness and mesenchymal phenotypes in breast and lung cancer (15,17) we here discovered that depletion of SIX2 reduces cell plasticity and stemness in AR-negative prostate cancer cells. This occurs through the direct regulation of stem cell programs, including Wnt signalling. Specifically, the silencing of SIX2 reduces nuclear β-catenin and Wnt/β-catenin signalling activity. Mechanistically, SIX2 binds directly to the promoter regions of key components of Wnt signalling pathway, such as Wnt ligands and Frizzled receptors, thereby inducing their expression. Previously, activation of WLS-Wnt signalling and FZD8 upregulation have reported to promote progression of treatment-induced neuroendocrine prostate cancer and prostate cancer metastasis (38–40). Consequently, the depletion of SIX2 results in reduced expression of these components and decreased activity of Wnt signalling leading to decreased cancer stemness. This reduction leads to decreased expression of Wnt/β-catenin activated target proteins c-Myc, MMP7, MET, Cyclin D1 and CD44 as well as known pluripotency regulators SOX2 and NANOG. Moreover, our findings demonstrate that SIX2 depletion blocks the ability of PC-3 cells to form colonies, indicating reduced stemness.

In this study, we reported that SIX2 has an important role in maintaining malignant properties of highly metastatic, androgen-independent PC-3 cells. SIX2 has previously been implicated in mediating late-stage metastasis in breast (16,17) and lung (15) cancers by regulating the pluripotency factor SOX2 and the epithelial marker E-cadherin expression. We found that depletion of SIX2 in PC-3 prostate cancer cells effectively prevented metastatic properties including proliferation, migration, invasion and colony formation ability. Additionally, SIX2 silencing significantly reduced invasion and formation of distant colonies of PC-3 cells in prostate cancer zebrafish *in vivo* models. Mechanistically, our results revealed that SIX2 silencing activated mesenchymal to epithelial transition by reducing the expression of Wnt/β-catenin activated target MMP7 as well as SNAIL and SLUG, which are transcriptional regulators of EMT and cancer metastasis (41–43).

To summarize, our study reports for the first time, the role for transcription factor SIX2 in modulating cell stemness and plasticity in prostate cancer. In AR-negative, treatment resistant phenotypes, the depletion of SIX2 reverses the phenotypic plasticity and stem cell lineage commitment associated with prostate cancer treatment resistance, establishing SIX2 as a novel regulator of these plastic phenotypes in prostate cancer. Our findings demonstrate that SIX2 silencing significantly reduces malignant properties, including migration, invasion, and colony formation ability of prostate cancer cells. Moreover, we identify SIX2 as an early responsive gene for ENZ that plays a pivotal role in mediating the subsequent expression of c-Myc, MMP7, Cyclin D1, CD44, SOX2 and NANOG among others in prostate cancer. Our study provides critical insights into how AR suppression induces the expression of SIX2 transcription factor and how the depletion of this potential phenotype and resistance-defining transcription factor targets plasticity programs, including stemness and Wnt signalling. Consequently, this depletion exhibits potent inhibitory effects on cell proliferation, colony formation, and metastasis both *in vitro* and *in vivo*. These findings emphasize the importance of developing novel means to target and/or suppress SIX2 in AR-negative prostate cancer tumors, aiming to target and/or prevent metastatic antiandrogen treatment-resistant phenotypes. However, as a transcription factor, direct inhibition of SIX2 may be challenging. Thus, understanding the regulation of SIX2 and its potential cofactors may be important for enabling the targeting of the SIX2-controlled transcriptional program in the future.

## Supplementary Material

gkae206_Supplemental_File

## Data Availability

The cBioPortal for Cancer Genomics is an open-access portal (http://cbioportal.org) that enables interactive, exploratory analysis of large-scale cancer genomics data. ATAC-seq, ChIP-seq and RNA-seq data has deposited to GEO; GEO accession: GSE235958.
