# Supplementary material for: SIX2 promotes cell plasticity via Wnt/β-catenin signalling in androgen receptor independent prostate cancer
Source: Nucleic Acids Res. 2024 Mar 30;52(10):5610–23. doi: 10.1093/nar/gkae206 (PMC11162805; doi:10.1093/nar/gkae206)
Supplement: gkae206_Supplemental_File [file gkae206_supplemental_file.pdf]

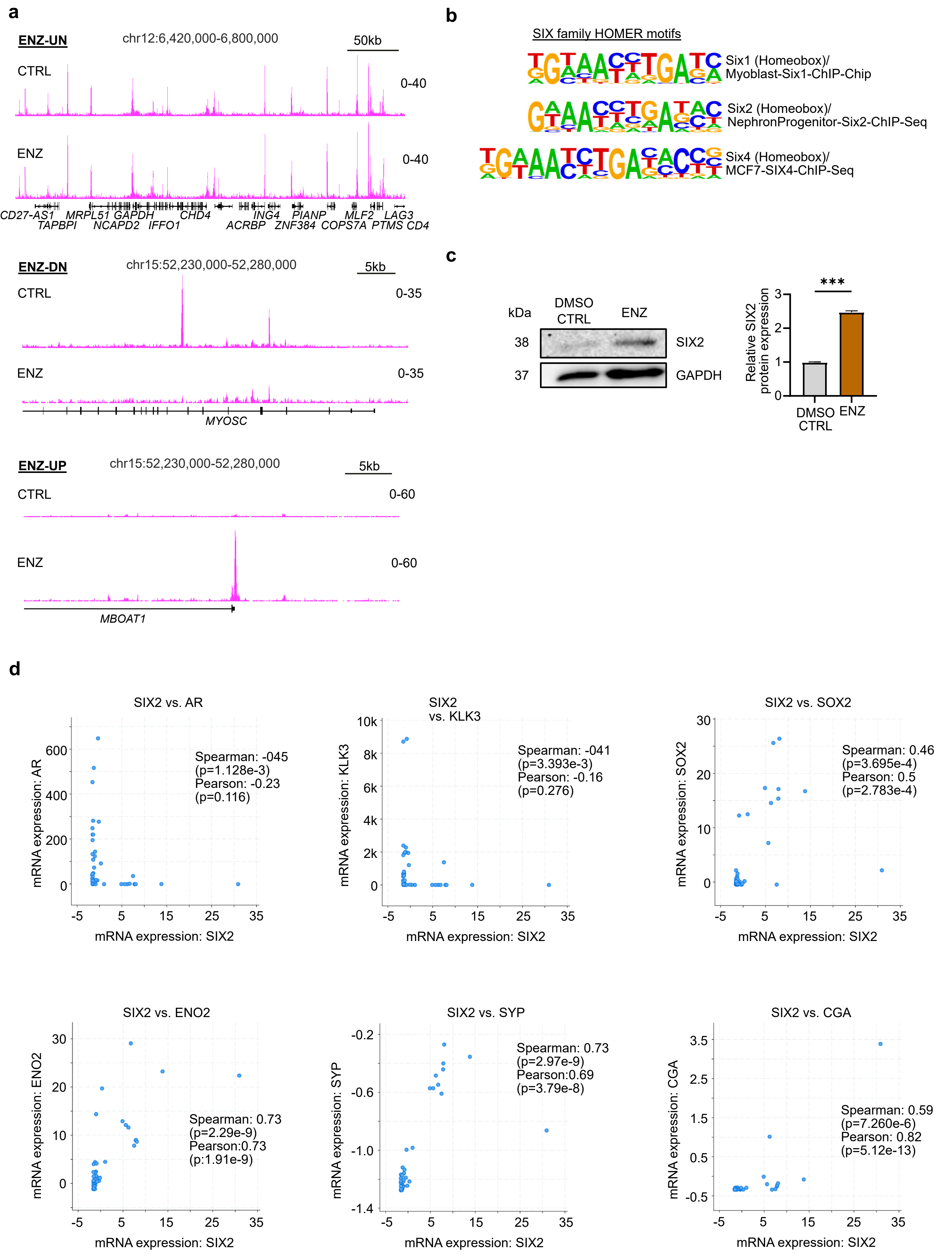

Supplementary Fig S1 - see next page for caption

Supplementary Figure S1

a Genome browser tracks from ATAC-seq of long-term ENZ exposed LNCaP cells representing chromatin regions unregulated, closed or opened by ENZ.

b HOMER motifs of SIX family proteins SIX1, SIX2 and SIX4.

c Western blot showing SIX2 protein expression in LNCaP cells exposed to AR inhibitor ENZ (left) and GAPDH normalized relative SIX2 protein expression (bottom).

d Co-expression plots and correlation values of *SIX2* and *AR, KLK3, SOX2, ENO2, SYP* and *CGA*, Data from cBioPortal, Beltran et al. 201 tracks from ATAC-seq of long-term ENZ exposed LNCaP cells representing chromatin regions unregulated, closed or opened 6 patient tumor samples.

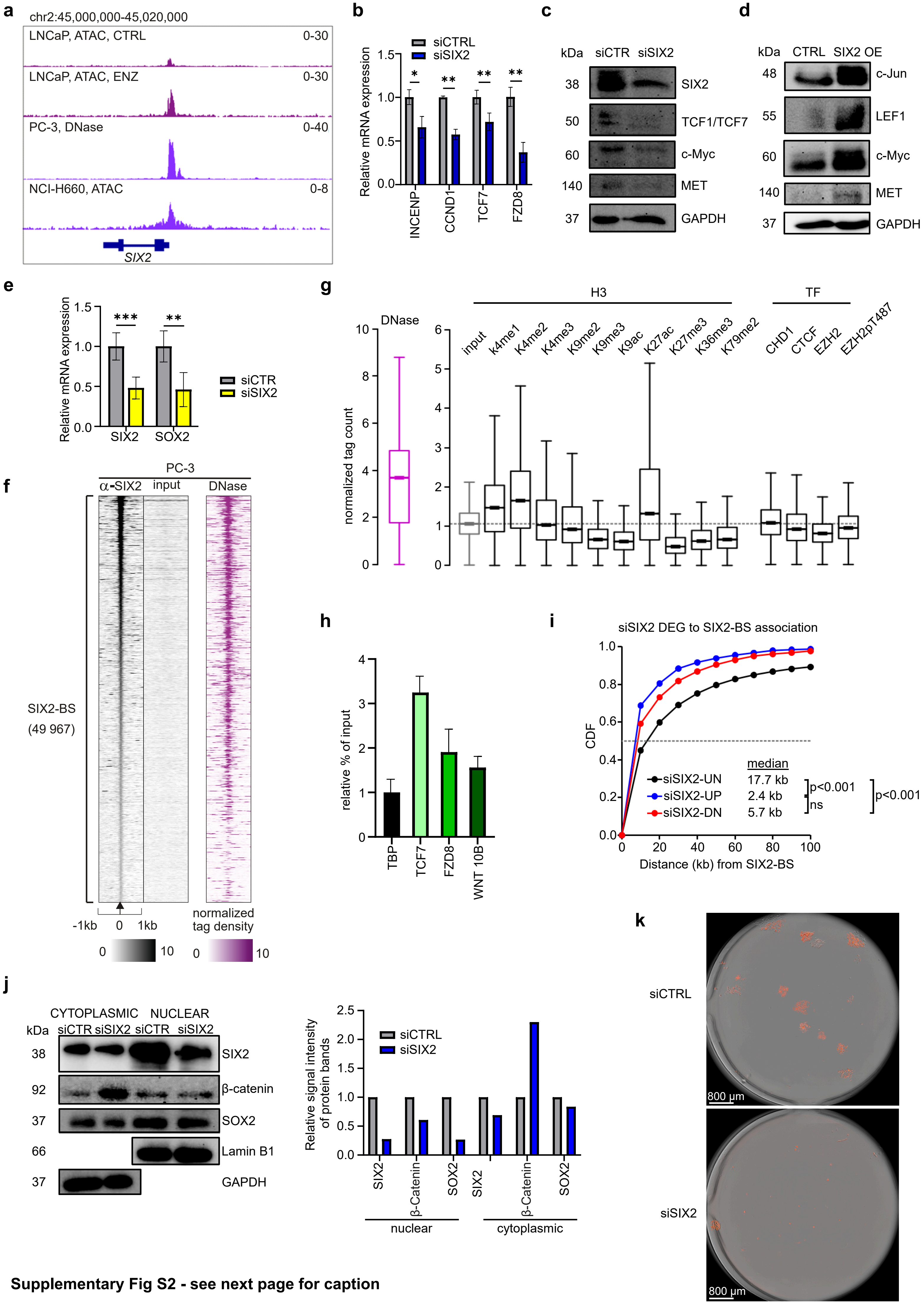

Supplementary Fig S2 - see next page for caption

Supplemental Figure S2

a SIX2 promoter is open and active in PC-3 and NCI-H660 cells similarly as the promoter openness is increased in LNCaP cells upon ENZ treatment. Comparison of our LNCaP ATAC-seq data with (ENZ) or without (CTRL) enzalutamide treatment to publicly available chromatin accessibility data from PC-3 (DNase-seq; ENCSR052AWE, ENCODE) and NCI-H660 (ATAC-seq; GSE118207, Park et al. 2018 Science) cells.

b Validation of RNA-seq results using qPCR analysis. Relative mRNA expression of genes downregulated by siSIX2.

c Western blot shows protein expression of Wnt/β-Catenin activated targets in siCTRL and siSIX2 NCI-H660 cells.

d Western blot shows protein expression of Wnt/β-Catenin activated targets in control and SIX2 overexpressed LNCaP cells.

e Relative *GAPDH* normalized *SIX2*, and *SOX* mRNA expression in control and SIX2 silenced NCI-H660 cells.

f Heatmap displaying SIX2 binding intensity in PC-3 cells. 49 967 peaks were called.

g Box blot representing enrichment of SIX2 binding sites compared to histone modifications and transcription factors.

h qPCR analysis validation of SIX2 ChIP-seq in PC-3 cells. TBP used as a negative control, (n=3).

i A cumulative distribution function (CDF) plot representing association of siSIX2 vs siCTRL differentially expressed genes and SIX2 binding sites in PC-3 cells.

j Western blot images of nuclear and cytoplasmic extract from control and SIX2 silenced NCI-H660 cells (left) and normalized expression of nuclear protein expressions in bar graph (right).

k Microscopy images of whole wells representing colony formation of siCTR and siSIX2 PC-3 cells with stable red fluorescence nuclear stain. Images with phase and red fluorescence taken by IncuCute on 9 days post plating cells.

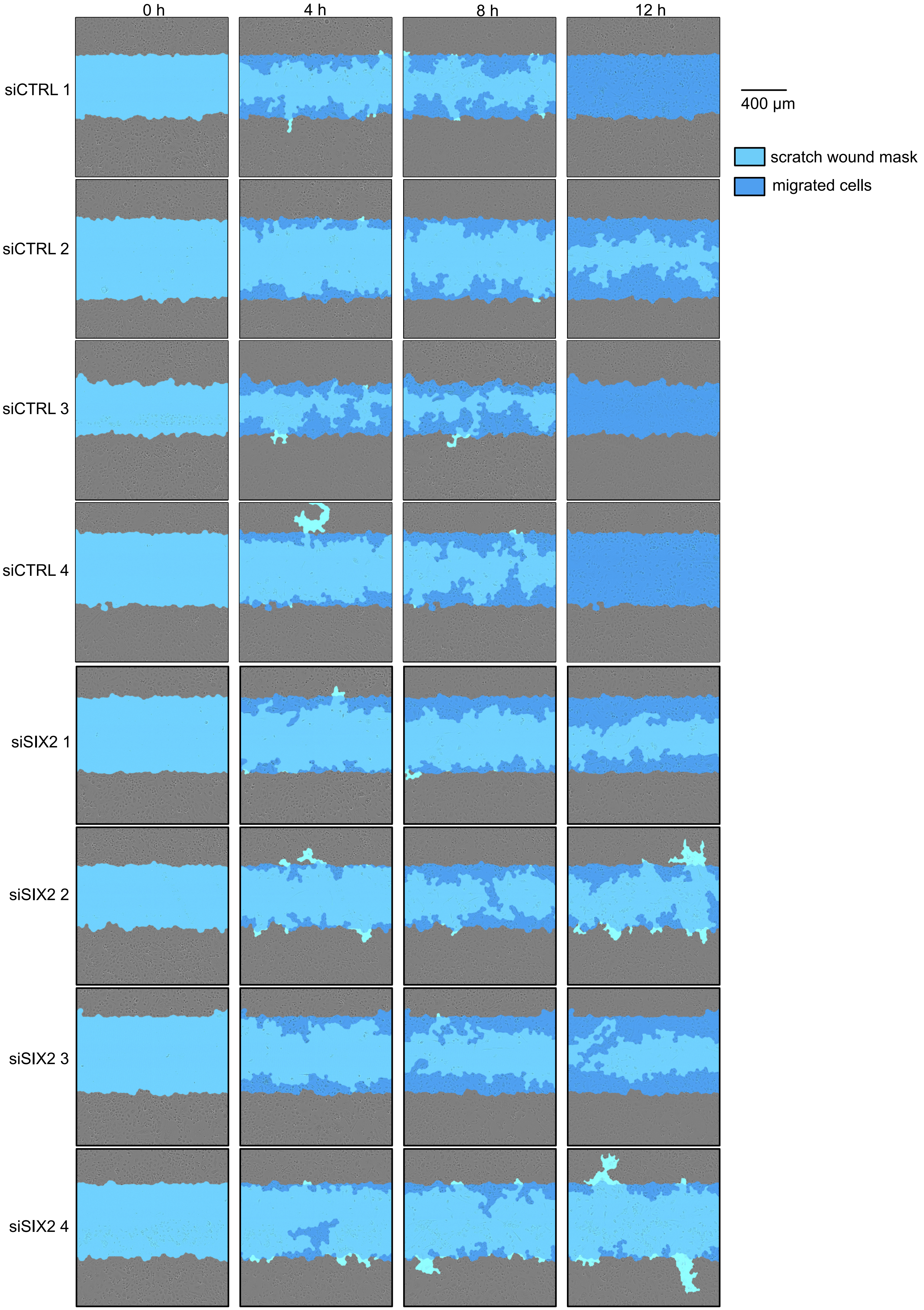

Supplementary Fig S3 - Example images from scratch wound assay of siCTRL and siSIX2 PC-3 cells at 0, 4, 8 and 12 hours post wound scratching. The scratch wound mask shown as cyan and migrated cells as blue.

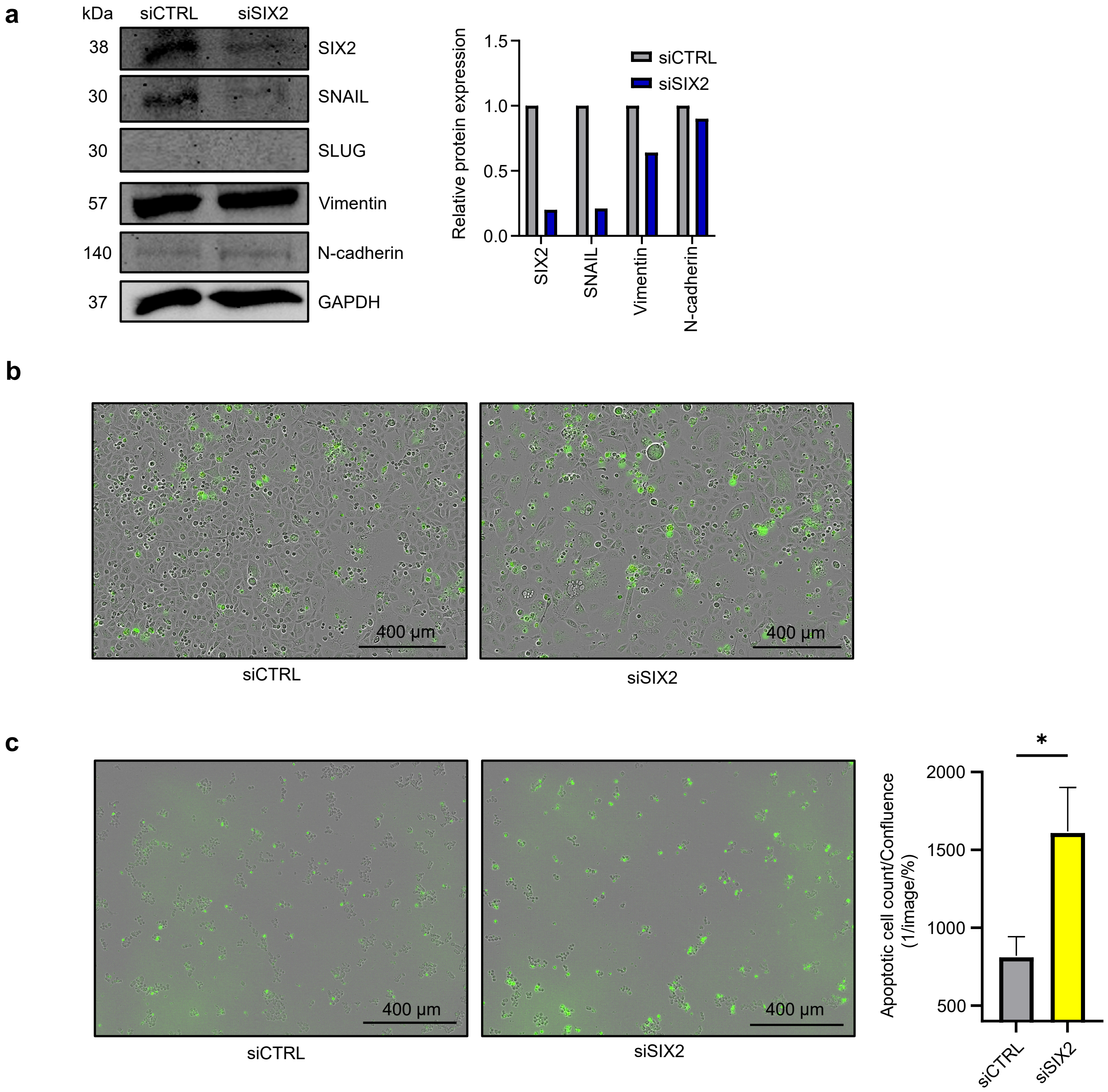

Supplementary Fig S4

a Silencing of SIX2 downregulates the protein expression of EMT regulator SNAIL and mesenchymal markers in PC-3 cell spheroids assessed using western blot. The quantification of the protein expression in siCTRL and siSIX2 samples in comparison to GAPDH loading control was calculated and shown on the left.

b The effects of siCTRL and siSIX2 on apoptotic PC-3 cells (green) assessed using IncuCyte S3 and Caspase 3/7 green Apoptosis reagent.

c Microscopy images showing the effects of siCTRL and siSIX2 on apoptotic NCI-H660 cells (green) assessed using IncuCyte S3 and Caspase 3/7 green Apoptosis reagent (left) and apoptotic cells (%) of control and SIX2 silenced NCI-H660 cells based on Caspase 3/7 staining in incucyte (n=6) (right).

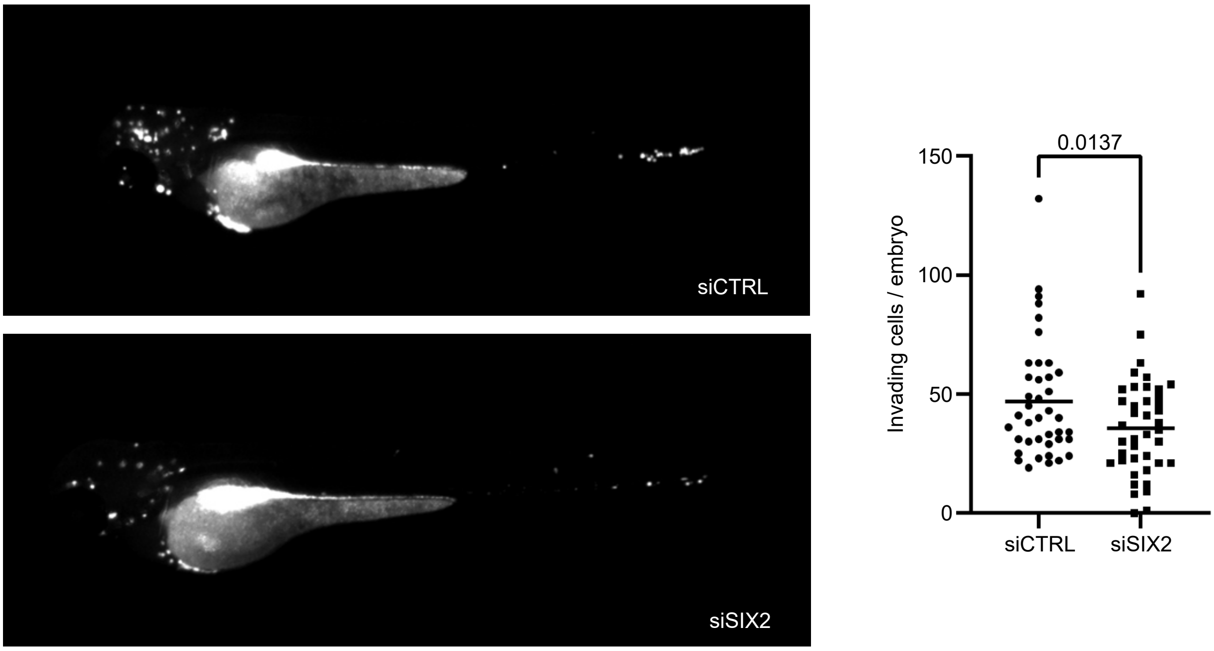

Supplementary Fig S5 Zebrafish intravascular dissemination experiment.

Images of zebrafish embryos 24h after intravascular transplantation of PC-3 siCTRL or siSIX2 cells expressing nuclear red fluorescent protein (left) and quantification of the number of invaded cells per embryo. The effect of siCTRL and siSIX2 on PC-3 invasion in the zebrafish vein dissemination analysis, mean marked as line. 1-sided t-test p-value 0.0137 (siCTRL n=40, siSIX2 n=41) (right).

Supplemental table S1: Antibodies used in immunoblotting

	Protein / Antibody	producer	catalog	dilution
primary antibodies	GAPDH	Invitrogen	# PA1-987	1:5000
	Lamin B1	Santa Cruz Biotechnology	# sc-374015	1:1000
	SIX2	Sigma-Aldrich	# HPA056958	1:1000
	SOX2	Cell Signaling	# D609	1:500
	Nanog	Abcam	# ab21624	1:500
	β-Catenin	Santa Cruz Biotechnology	# sc-7963	1:1000
	LEF1	Cell Signaling	#2230	1:1000
	TCF1/TCF7	Cell Signaling	# 2203	1:1000
	c-Jun	Cell Signaling	# 9165	1:1000
	c-Myc	Cell Signaling	# 5605	1:1000
	Cyclin D1	Cell Signaling	# 2978	1:1000
	MMP7	Cell Signaling	# 3801	1:1000
	MET	Cell Signaling	# 8198	1:1000
	CD44	Cell Signaling	# 3570	1:1000
	SNAIL	Cell Signaling	# 3879	1:1000
	SLUG	Cell Signaling	# 9585	1:1000
	Vimentin	Cell Signaling	# 5741	1:1000
	Claudin-1	Cell Signaling	# 13255	1:1000
	E-cadherin	Cell Signaling	# 3195	1:1000
	N-cadherin	Cell Signaling	# 13116	1:1000
secondary antibodies	goat anti-rabbit IgG (H+L)	Invitrogen	# G-21234	1:10000
	goat anti-mouse IgG (H+L)	Invitrogen	# G-21040	1:10000

Supplemental Table S2. Primers used in RT-qPCR experiments

Gene name	Forward (5' to 3')	Reverse (5' to 3')
GAPDH	CGACCTGACCTGCCGTCTAGAA	GGTGTCGCTGGTGAAGTCGAGAG
SIX2	CACACAGGTCAGCAACTGGTTC	TCATCCTCCGAGCTGCCTAACA
NANOG	AGTCCCAAAGGCAAACAACCCACTTC	TGCTGGAGGCTGAGGTATTTCTGTCTC
SOX2	TGCGAGCGCTGCACA	TCATGAGCGTCTTGGTTTTCC
CD44	AGTACAACGGAAGAAACAGC	TTGGGTTGAAGAAATCAGTC
INCENP	AGGCTCCTGAATGTTGAGGTGC	GTGTGCTGTTGGCAATCTCCGT
TCF7	CTGCCATCAACCAGATCCT	GCTCATAGTACTTGGCCTGCT
FZD8	GCTCTACAACCGCGTCAAGA	GCTGAAAAAGGGGTTGTGGC
CCND1	GCTGTGCATCTACACCGACA	TTGAGCTTGTTCACCAGGAG
TBP	GGGGAGCTGTGATGTGAAGT	GAGCCATTACGTCGTCTTCC
SIX2-ChIP *TCF7*	AGGCCGAGGGATTCTGACTA	AGTCCCAGGCTTTCATGTGG
SIX2-ChIP *FZD8*	TTTATACAGCGCGGACTCGG	ACACTCGGGGACTGCCTTA
SIX2-ChIP *WNT10b*	TTACCTCCAGTGGTTTGGGTG	CCCACGGTTTAAGCAGCACT
